# Position-wise comparison of handheld 6-lead ECG versus the standard 12-lead ECG in patients with arrhythmia: comparative study

**DOI:** 10.1186/s12872-026-05971-x

**Published:** 2026-06-08

**Authors:** Young Jun Park, Sujeong Eom, Sang Jun Lee, Yeongyeon Na, Jeonghwa Lim, Minje Park, Hyun Jin Ahn, Mineok Chang, Sunghoon Joo, Min-Soo Ahn

**Affiliations:** 1 Division of Cardiology, Department of Internal Medicine, Korea University College of Medicine and Korea University Anam Hospital, Seoul, Republic of Korea; 2 VUNO Inc., Seoul, Republic of Korea; 3 Division of Cardiology, Department of Internal Medicine, Wonju Severance Christian Hospital, Yonsei University Wonju College of Medicine, Wonju, Republic of Korea

**Keywords:** Handheld 6‑lead ECG, 12‑lead ECG, Arrhythmia, Signal equivalence

## Abstract

**Background:**

As 12‑lead electrocardiographs (ECGs) require a clinical infrastructure that limits timely access, portable 6‑lead devices may extend diagnostics to community and remote settings. We evaluated the signal equivalence of a handheld 6‑lead ECG (HATIV^®^ P30) versus the standard 12‑lead in an arrhythmia cohort, considering posture and synchrony.

**Methods:**

In this prospective single-center study, simultaneous 10-s 12-lead ECGs and time-aligned 10-s segments from 30-s 6-lead recordings were obtained from arrhythmia patients in both supine and sitting positions. A blinded electrophysiologist performed rhythm classification and ECG measurements. Diagnostic accuracy and numerical agreement of key parameters (PR interval, QRS duration, QT/QTc intervals, and amplitudes) were evaluated.

**Results:**

A total of 229 paired recordings were analyzed after excluding 6 pairs. The overall diagnostic accuracy of the 6‑lead versus 12‑lead was 99.1% in the supine position (*n* = 113) and 99.1% in the sitting position (*n* = 116); one atrial flutter was misclassified as atrial fibrillation in each position. Bland–Altman analyses showed small mean differences (12‑lead minus 6‑lead): PR + 12.1/ + 7.4 ms (supine/sitting), QRS − 6.4/ − 6.0 ms, QT − 10.3/ − 5.3 ms, QTc − 11.5/ − 6.4 ms; heart‑rate difference ≈0.03 bpm. The absolute differences were < 20 ms in approximately ~ 70% for PR and ~ 55–62% for QT/QTc. In an exploratory asynchronous pairing (supine 12‑lead vs sitting 6‑lead; *n* = 103), accuracy decreased to 97.1% and parameter differences widened, consistent with postural/temporal effects.

**Conclusions:**

In patients with arrhythmia, the handheld 6‑lead showed near‑perfect rhythm agreement and small numerical differences versus the 12‑lead under synchronized acquisition in both positions. Asynchronous or posture-mismatched comparisons reduce the agreement, and acquisition conditions should be considered. The 6‑lead may be a practical alternative when the 12‑lead is unavailable in patients with arrhythmia.

**Supplementary Information:**

The online version contains supplementary material available at 10.1186/s12872-026-05971-x.

## Introduction

Electrocardiography (ECG) remains the gold standard for detecting and managing cardiac abnormalities. However, conventional 12-lead ECGs require dedicated clinical infrastructure and equipment, thereby limiting timely access to diagnostic services, particularly in community or remote settings, where such facilities are often unavailable. The emergence of portable ECG technologies, including 6-lead and single-lead consumer-based monitors, has generated considerable interest as potential adjuncts to standard ECG systems [1]. However, before these devices can be adopted in broader clinical workflows or extended to community settings, thorough validation of their signal equivalence against the 12-lead standard is essential.

Recent studies have increasingly evaluated the diagnostic performance and numerical agreement of handheld 6-lead ECG devices compared to the standard 12-lead ECG. For atrial fibrillation (AF) detection, 6-lead devices have demonstrated high diagnostic accuracy (sensitivity 99%), outperforming their single-lead counterparts [2–4]. While earlier meta-analyses—largely focused on single-lead technology—reported sensitivities of 89% to 92%, the emergence of multi-lead configuration has further enhanced diagnostic reliability [1]. Similarly, in the monitoring of QT intervals, a critical parameter associated with drug safety, 6-lead devices have shown strong performance, with a reported negative predictive value of 99.8% for detecting QTc prolongation beyond 500 ms [5]. More broadly, 6-lead ECGs have demonstrated clinical utility across a variety of populations, including patients with general cardiologic conditions, tuberculosis, genetic heart disease, athletes and pediatric populations [5–10]. These findings support the potential clinical utility of 6-lead devices in diverse settings.

However, the strength of this evidence depends on the rigor of the validation methodology employed, and important gaps remain. In particular, differences in patient posture during acquisition—supine for 12-lead and typically sitting for 6-lead ECGs—may influence signal morphology and diagnostic interpretation but are often overlooked. Specifically, recent studies have indicated that variations in patient positioning across devices may lead to changes in ECG parameters, including QRS morphology and heart rate, thereby posing potential limitations [7, 9, 11]. Additionally, most previous studies involved non-simultaneous recordings of 12-lead and 6-lead ECGs, introducing temporal discrepancies that limit direct comparability. Moreover, 6-lead ECG devices have not been extensively evaluated in populations with a high burden of arrhythmia. These methodological limitations make it difficult to determine whether observed discrepancies reflect true device differences or are artifacts of acquisition conditions. Comprehensive assessment of both rhythm classification accuracy and numerical agreement of ECG parameters within a single study is also uncommon.

This study was designed as a controlled clinical validation to rigorously assess diagnostic accuracy and numerical agreement of the handheld 6-lead ECG device, HATIV^®^ P30 (VUNO Inc., 479, Gangnam-daero, Seocho-gu, Seoul, Republic of Korea, 06541), against the 12-lead standard under standardized acquisition conditions. Using simultaneous recordings in both supine and sitting positions within an arrhythmia-enriched cohort, we aimed to isolate intrinsic device differences from confounding effects of temporal and postural variability. To our knowledge, this is the first study to evaluate both positional effects and simultaneous acquisition in such a population. The primary objective was to assess the signal equivalence between the two devices in terms of rhythm interpretation and key ECG parameters, including PR interval, QRS duration, QT/QTc intervals and amplitudes. These findings provide a foundation for future evaluation in less controlled settings, such as ambulatory and community environments.

## Methods

### Study design and population

This prospective, single-center, observational study was conducted at Wonju Severance Christian Hospital, a tertiary academic medical center in South Korea, between October 2022 and August 2023. The study aimed to evaluate the signal equivalence and diagnostic reliability of a handheld 6-lead ECG device (HATIV^®^ P30, VUNO Inc., Seoul, Republic of Korea), (hereinafter referred to as HATIV), compared to the conventional 12-lead ECG system (MAC 2000, GE Healthcare, WI, USA), which is considered the clinical gold standard. We evaluated the comparability in terms of rhythm interpretation and signal parameter accuracy.

Eligible participants were adults aged 19–80 years who visited or were admitted to the cardiology department with clinically documented arrhythmias. Consecutive patients who met the inclusion criteria were prospectively enrolled. However, due to the prospective study design and practical constraints in clinical settings (e.g., patient refusal or limited research staff availability), not all eligible patients could be enrolled during the study period. All participants were required to provide written informed consent, demonstrate their ability to understand the study instructions, and comply with the procedures. Participants were excluded if they received an implanted intracardiac device, following HATIV’s regulatory labeling (Ministry of Food and Drug Safety of Korea).

Baseline characteristics, including demographics (age, sex, and body surface area [BSA]), comorbidities (hypertension, diabetes, and dyslipidemia etc.), and documented arrhythmia type were collected.

### Electrocardiogram acquisition

The handheld 6-lead ECG devices used in this study were provided by the manufacturer (VUNO Inc.). However, the study was conducted independently at the clinical site. The manufacturer had no role in patient recruitment, data acquisition, or outcome assessment.

**Fig. 1:**
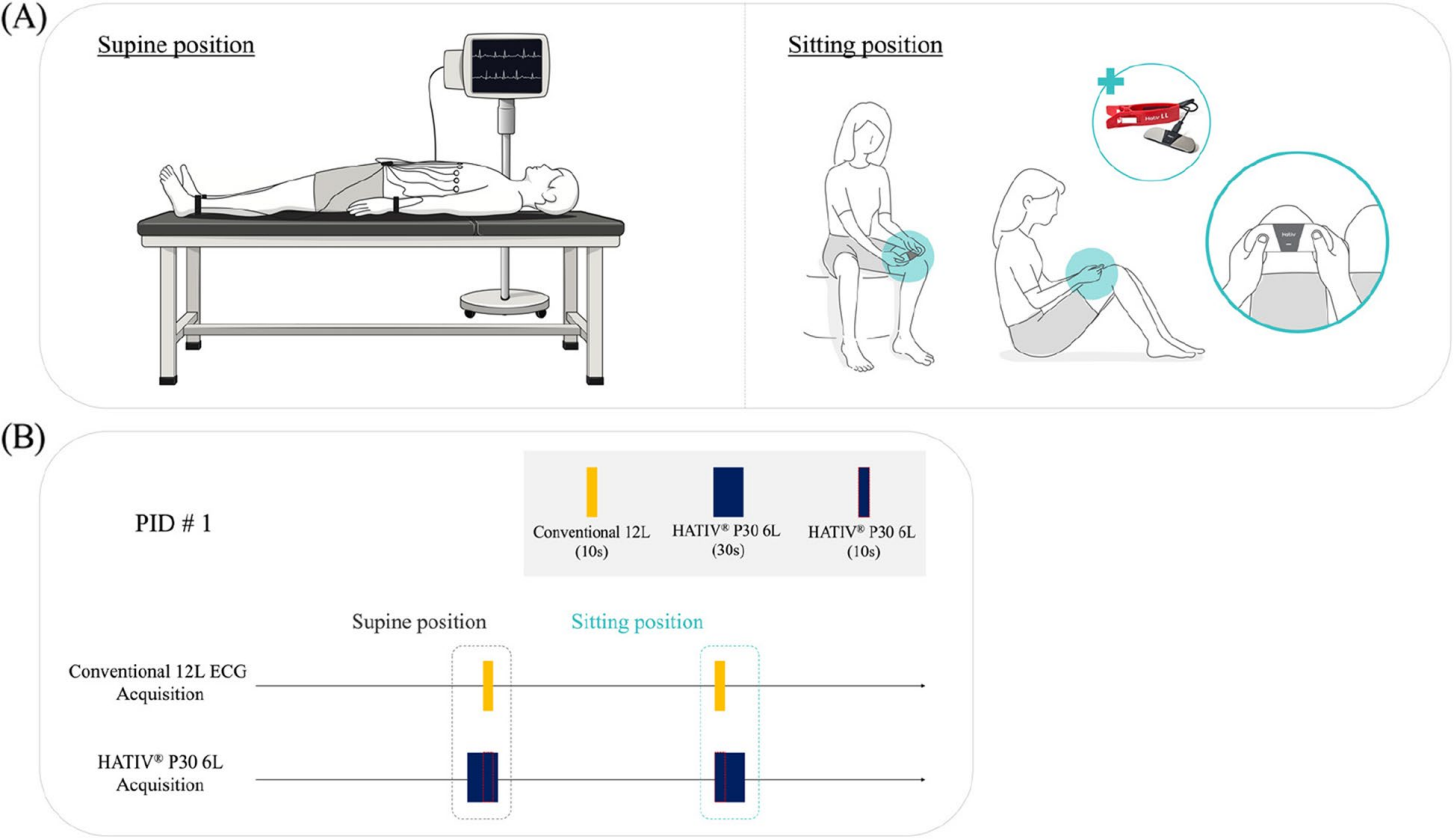
Simultaneous acquisition and synchronous pairing of 12-lead and 6-lead ECGs. **A** Illustrations of the standard acquisition positions for each device: 12-lead ECG in the supine position and 6-lead ECG in the sitting position. In the supine position, the 6-lead device was held with both hands, and the left leg electrode was simulated by attaching an additional electrode to the left ankle (highlighted in red). **B** In this study, to account for positional effects that may influence signal morphology, both 12-lead and 6-lead ECGs were recorded in both supine and sitting positions. Although the HATIV device records a continuous 30-s 6-lead ECG, only the 10-s segment temporally aligned with the 10-s 12-lead ECG was used for synchronous analysis

To enable a precise comparison, 12-lead and 6-lead ECGs were acquired simultaneously from each participant. All the ECG recordings were performed by a single trained nurse. The standard 10-s 12-lead ECG was acquired using the MAC 2000 system [12]. The 30-s 6-lead ECG was recorded using the HATIV, which utilizes three contact points to derive six frontal plane leads (I, II, III, aVR, aVL, and aVF). The device incorporates embedded electrodes for both thumbs (corresponding to the left and right arms) and a third contact at the left ankle or thigh to approximate the left leg electrode. Generally, a 12-lead ECG is obtained in the supine position, whereas a 6-lead ECG is obtained in the sitting position (Fig. 1a). However, to account for positional effects that may influence signal morphology, we recorded ECGs from both devices in both the supine and sitting positions. In the supine position, the 6-lead device was held with both hands, and the left leg electrode position was simulated by attaching an additional electrode to the left ankle. This dual-position design ensured that the inter-device comparisons were not confounded by postural variability.

The 12-lead ECG samples signals at 500 Hz, and the 6-lead ECG samples at 250 Hz. The bandpass filter specifications for both devices were 1–40 Hz. All recordings were digitally stored upon acquisition, using the proprietary system of each device. After recording, all ECG files were uploaded to a custom web-based labelling platform that enabled visualization and annotation.

### Expert annotation

Although the HATIV records a continuous 30-s 6-lead ECG, only the 10-s segment that was aligned on exact recording timestamps with the 12-lead recording was used for annotation (synchronous pairing; Fig. 1b).

**Fig. 2:**
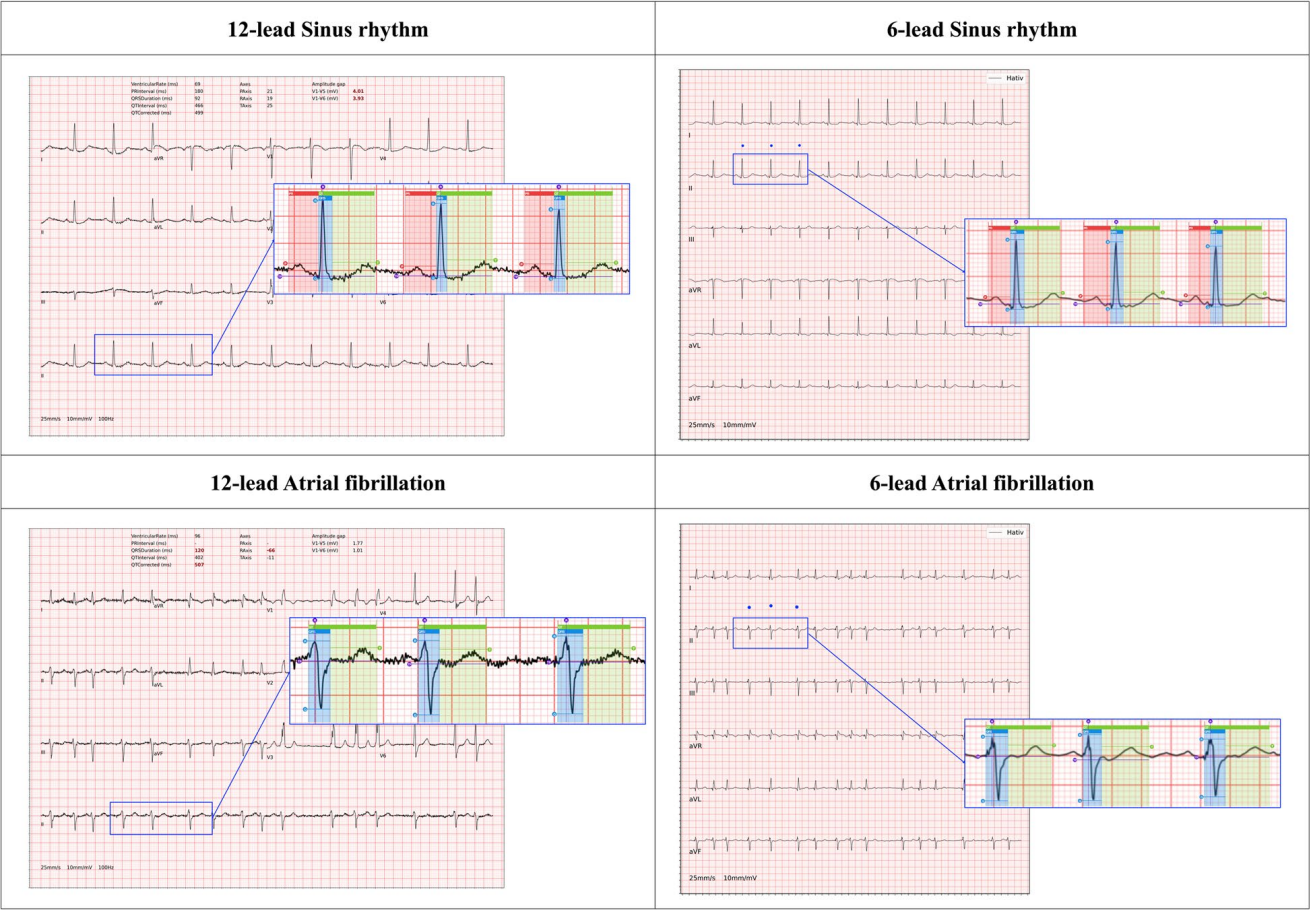
Representative examples of beat labelling on lead II for ECG parameter annotation. Paired examples of 12-lead and 6-lead ECGs in sinus rhythm and atrial fibrillation are shown. Each 10-s ECG recording was independently reviewed by a rater for rhythm classification. Three representative beats are selected and annotated. The horizontal purple line represents the isoelectric baseline. In both cases, the three beats were first labelled on the 12-lead ECG, and the corresponding temporally aligned beats (marked with blue dots) were subsequently labelled on the 6-lead ECG. ECG, electrocardiogram

A single board-certified electrophysiologist with > 10 years of clinical experience interpreted both ECGs in a blinded manner. Rhythm interpretation and manual beat labelling were performed per ECG. For rhythm interpretation, each ECG was evaluated for the presence of sinus rhythm, atrial premature complex (APC), ventricular premature complex (VPC), atrial fibrillation (AF), and atrial flutter (AFL). Beat labelling included the placement of fiducial points (P, QRS, and T) on three representative and noise-free beats of lead II. The annotator was instructed to label the representative beats consistent with the rhythm category. The intervals (PR interval, QRS duration, QT interval, and heart rate-corrected QT [QTc] interval; ms) and amplitudes (P amplitude, QRS amplitude, and T wave; mV) were measured (Fig. 2). QTc interval was calculated using the Bazett formula. Heart rate (beats per minute, bpm) was calculated based on a 10-s ECG recording.

Furthermore, to minimize labelling bias, in half of the cases, a 12-lead ECG was first provided for beat labelling, followed by a 6-lead ECG with the corresponding beats marked. In the other half, labelling began with the 6-lead ECG and was subsequently applied to the 12-lead ECG. To ensure the consistency of the annotations, any discrepant cases or outliers in the Bland–Altman analysis were reviewed a second time by the same reader.

### Statistical analysis

Clinical characteristics are summarized as continuous variables presented as means with standard deviations (SD), and categorical variables are presented as numbers with percentages. All synchronous pairing analyses were performed in both supine and sitting positions.

For rhythm interpretation, 12-lead ECG was used as the reference diagnosis. The diagnostic performance of the 6-lead ECG was evaluated for accuracy, sensitivity, specificity, positive predictive value (PPV), and negative predictive value (NPV), each accompanied by the corresponding 95% confidence intervals (CI) for each rhythm category. Confidence intervals were calculated using the Clopper-Pearson method [13]. ECG pairs with ungradable 12-lead recordings were excluded because the 12-lead ECG was used as the reference standard.

The numerical agreement between 12-lead and 6-lead ECG parameters (PR interval, QRS duration, etc.) was assessed. Noisy recordings were excluded from the analyses. When a P wave was absent in one or more beats, such as in AF, the corresponding ECG was excluded from the PR interval and P amplitude comparisons. For each ECG parameter obtained from beat labelling, the average across three beats was calculated and used as the representative value for that ECG. Descriptive summary statistics were calculated using means (SD). Paired comparisons between the 12-lead and 6-lead ECGs were conducted using the Bland–Altman method [14]. This method estimates the mean difference and limits of agreement (LOA) to quantify the degree of signal agreement and potential bias. Differences were calculated as 12-lead minus 6-lead values; thus, negative values indicated higher measurements from the 6-lead ECG. Absolute differences were further analyzed in categories, presenting counts and percentages for defined thresholds (e.g. interval absolute differences < 20 ms).

An “ungradable ECG” was defined as a recording in which rhythm interpretation was not possible. In contrast, “noisy recordings” refer to ECGs in which reliable beat-level annotation (e.g., identification of fiducial points) was not feasible, and these were excluded only from the numerical agreement analysis. All analyses were conducted according to a predefined protocol using anonymized data.

### Exploratory analysis

Two additional analyses were performed to further explore signal comparability under various conditions. First, the numerical agreement specifically for AF ECGs was assessed using absolute differences. Given the inherently high variability in AF signals, we investigated whether the differences between 12-lead and 6-lead ECGs widened in AF cases. Second, an asynchronous pairing analysis was conducted. For exploratory validation, non-simultaneous ECGs were matched post hoc by pairing a supine 12-lead ECG with a sitting 6-lead ECG from the same patient, reflecting the standard acquisition posture for each device (Supplementary Figure S1). The comparisons were restricted to pairs with consistent rhythm classifications for both devices. Rhythms that may be affected by premature complexes (APC and VPC) were grouped into sinus rhythm category for analysis.

All statistical analyses were performed using Python (version 3.11; Python Software Foundation, Wilmington, DE, USA) with SciPy (version 1.12; SciPy Developers, Austin, TX, USA) library.

### Sample size

The sample size was determined to ensure adequate precision of the LOA in a Bland–Altman analysis [14]. Using data from a reference study reporting a mean difference of 0.62 ms and a SD of paired differences of 26.82 ms (12-lead vs. 6-lead), we estimated that a minimum of 83 participants would be sufficient to provide a reasonably precise estimate of the LOA [2]. To account for a potential attrition rate of 20%, the target sample size was set at 104 participants.

## Results

### Study population

**Fig. 3:**
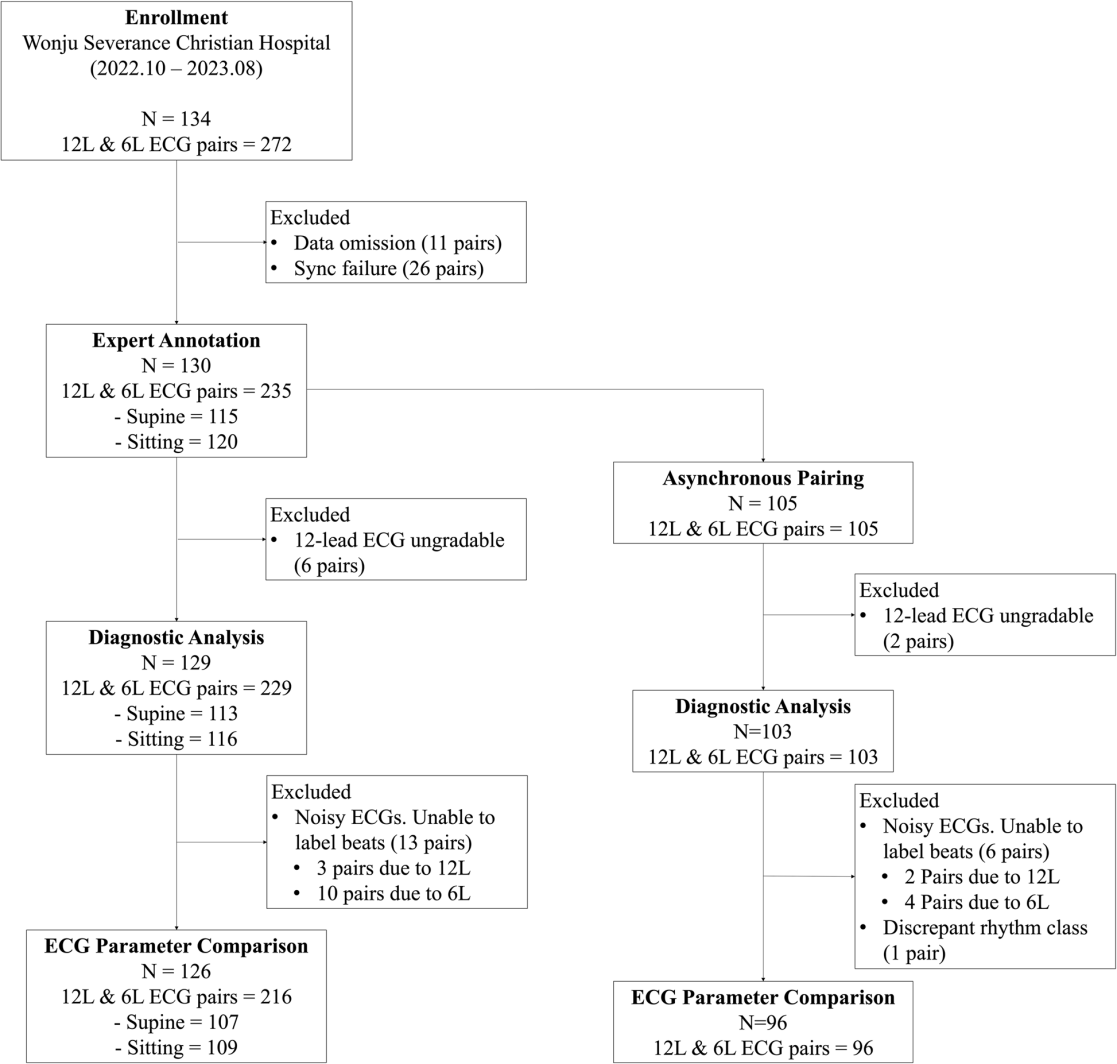
Study population and data flowchart. A total of 235 paired 12-lead and 6-lead ECGs were obtained from 130 patients, including 115 pairs in the supine position and 120 pairs in the sitting position. None of the patients underwent multiple recordings in the same position. For exploratory analysis, 12-lead ECGs acquired in the supine position were matched post hoc with 6-lead ECGs acquired in the sitting position to evaluate the postural effects. An “ungradable ECG” was defined as a recording in which rhythm interpretation was not possible. In contrast, “noisy recordings” refer to ECGs in which reliable beat-level annotation (e.g., identification of fiducial points) was not feasible, and these were excluded only from the numerical agreement analysis. ECG, electrocardiogram

In total, 134 patients were enrolled in this study. After excluding the patients with incomplete ECG pairs, 235 pairs were annotated. Figure 3 illustrates the detailed flow of data for each analysis. The mean age of the study population was 64.7 years, and 63.1% were men. Hypertension (46.9%) and heart failure (26.9%) were the most common comorbidities. The type of arrhythmias recorded was atrial fibrillation, which predominated (63.1%; paroxysmal 33.1%, persistent 30.0%), followed by premature beats (33.8%) and AFL (17.7%), whereas advanced atrioventricular block (1.5%) was relatively rare (Table 1).

**Table 1:** Demographics of study population

Demographic data	*N* = 130
Age	64.7 ± 11.1
Male	82 (63.1%)
BSA (m^2^)	1.8 ± 0.2
Hypertension	61 (46.9%)
Diabetes	32 (24.6%)
Dyslipidemia	27 (20.8%)
Coronary artery disease	14 (10.8%)
Heart failure	35 (26.9%)
Chronic kidney disease	4 (3.1%)
Cerebral infarction	11 (8.5%)
Cardiac surgery	3 (2.3%)
Arrhythmia type	
Atrial fibrillation	82 (63.1%)
Paroxysmal	43 (33.1%)
Persistent	39 (30.0%)
Atrial flutter	23 (17.7%)
Premature beats	44 (33.8%)
Atrial	11 (8.5%)
Ventricular	33 (25.4%)
Atrioventricular block	2 (1.5%)

### Diagnostic performance of 6-lead ECG in different positions

Six ECG pairs were excluded from the analysis, because the reference 12-lead ECGs were annotated as ungradable. The rhythm distributions in the 12-lead ECG included 93 sinus rhythms, 12 APCs, 32 VPCs, 74 AF, and 18 AFL. The HATIV demonstrated high concordance with the 12-lead reference in both supine and sitting positions. Overall diagnostic accuracy of the 6-lead compared to the 12-lead was 99.1% (95% CI, 95.2–100.0) in the supine position and 99.1% (95.3–100.0) in the sitting position. In both positions, AFL was misclassified as AF in one case (Table 2).

**Table 2:** Diagnostic performance of the handheld 6-lead ECG compared with the standard 12-lead ECG in different acquisition positions

Supine position (113 pairs)						
	**MUSE (N)**	**Accuracy** **(95% CI)**	**Sensitivity** **(95% CI)**	**Specificity** **(95% CI)**	**PPV (95% CI)**	**NPV (95% CI)**
Sinus rhythm	46	100.0 (96.8–100.0)	100.0 (92.3–100.0)	100.0 (94.6–100.0)	100.0 (92.3–100.0)	100.0 (94.6–100.0)
APC	7	100.0 (96.8–100.0)	100.0 (59.0–100.0)	100.0 (96.6–100.0)	100.0 (59.0–100.0)	100.0 (96.6–100.0)
VPC	15	100.0 (96.8–100.0)	100.0 (78.2–100.0)	100.0 (96.3–100.0)	100.0 (78.2–100.0)	100.0 (96.3–100.0)
AF	35	99.1 (95.2–100.0)	100.0 (90.0–100.0)	98.7 (93.1–100.0)	97.2 (85.5–99.9)	100.0 (95.3–100.0)
AFL	10	99.1 (95.2–100.0)	90.0 (55.5–99.7)	100.0 (96.5–100.0)	100.0 (66.4–100.0)	99.0 (94.8–100.0)

Sitting position (116 pairs)						
	**MUSE (N)**	**Accuracy** **(95% CI)**	**Sensitivity** **(95% CI)**	**Specificity** **(95% CI)**	**PPV (95% CI)**	**NPV (95% CI)**
Sinus rhythm	47	100.0 (96.9–100.0)	100.0 (92.5–100.0)	100.0 (94.8–100.0)	100.0 (92.5–100.0)	100.0 (94.8–100.0)
APC	5	100.0 (96.9–100.0)	100.0 (47.8–100.0)	100.0 (96.7–100.0)	100.0 (47.8–100.0)	100.0 (96.7–100.0)
VPC	17	100.0 (96.9–100.0)	100.0 (80.5–100.0)	100.0 (96.3–100.0)	100.0 (80.5–100.0)	100.0 (96.3–100.0)
AF	39	99.1 (95.3–100.0)	100.0 (91.0–100.0)	98.7 (93.0–100.0)	97.5 (86.8–99.9)	100.0 (95.3–100.0)
AFL	8	99.1 (95.3–100.0)	87.5 (47.3–99.7)	100.0 (96.6–100.0)	100.0 (59.0–100.0)	99.1 (95–100.0)

*ECG* electrocardiogram, *TP* true positive, *FP* false positive, *FN* false negative, *CI* confidence interval, *PPV* positive predictive value, *NPV* negative predictive value, *APC* atrial premature complex, *VPC* ventricular premature complex, *AF* atrial fibrillation, *AFL* atrial flutter

### Numerical agreement of 6-lead ECG in different positions

An additional 13 ECG pairs were excluded from the numerical agreement analysis because excessive noise in either the 12-lead (3 cases) or 6-lead (10 cases) ECG prevented beat labelling. In the supine position, 63 of the 107 pairs were included in the P-wave analysis, and 61 of the 109 pairs were included in the sitting position. The mean values of each parameter for both devices, including the asynchronous setting, are presented in Supplementary Table S1.

**Fig. 4:**
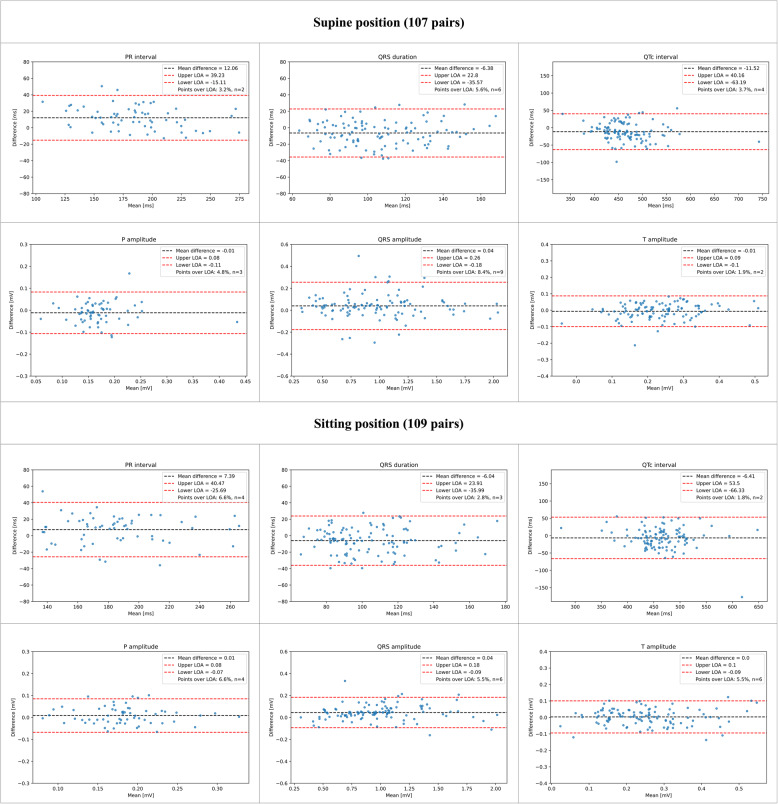
Bland–Altman plots comparing ECG parameters between 12-lead and 6-lead ECG. Negative values indicated higher measurements from the 6-lead ECG. LOA was calculated using the mean difference and SD. The upper LOA was defined as the mean difference plus 1.96*SD, and the lower LOA was defined as the mean difference minus 1.96*SD. Cases without identifiable P waves were excluded from PR interval and P amplitude analyses. ECG, electrocardiogram; LOA, limits of agreement; SD, standard deviation; ms, milliseconds; mV, millivolts

Table 3 and Fig. 4 present the results of the paired Bland–Altman analyses for each ECG parameter across different acquisition positions. The mean differences between the 12-lead and 6-lead ECGs for all parameters were small, supporting adequate rhythm classification in both positions. The mean difference in heart rate between the 12-lead and 6-lead ECGs was minimal, at 0.03 bpm for both the supine and sitting positions. The PR interval was longer in the 12-lead ECG in both positions (supine: mean difference 12.06 ms [LOA, − 15.11 to 39.23 ms]; sitting: 7.39 ms [− 25.69 to 40.47 ms]), whereas the QRS duration and QT/QTc intervals were longer in the 6-lead ECG. The mean difference in PR interval decreased from 12.06 ms (supine) to 7.39 ms (sitting), and in QT/QTc intervals from − 10.34/− 11.52 ms (supine) to − 5.32/− 6.41 ms (sitting); however, the LOA ranges widened in the sitting position for both parameters. QRS amplitude was consistently larger in the 12-lead ECG (supine: mean difference 0.04 mV [LOA, − 0.18 to 0.26 mV]; sitting: 0.04 mV [− 0.09 to 0.18 mV]).

**Table 3:** Bland–Altman analysis between the standard 12-lead and handheld 6-lead ECGs in different acquisition positions

Supine position (107 pairs)						
Metric	**N**	**Mean difference**	**SD**	**Upper LOA**	**Lower LOA**	**Outliers N (%)**
Heart rate (bpm)	107	0.03	1.12	2.23	− 2.17	8 (7.5)
PR interval (ms)	63	12.06	13.86	39.23	− 15.11	2 (3.2)
QRS duration (ms)	107	− 6.38	14.89	22.80	− 35.57	6 (5.6)
QT interval (ms)	107	− 10.34	22.68	34.11	− 54.79	6 (5.6)
QTc interval (ms)	107	− 11.52	26.36	40.16	− 63.19	4 (3.7)
P amplitude (mV)	63	− 0.01	0.05	0.08	− 0.11	3 (4.8)
QRS amplitude (mV)	107	0.04	0.11	0.26	− 0.18	9 (8.4)
T amplitude (mV)	107	− 0.01	0.05	0.09	− 0.10	2 (1.9)

Sitting position (109 pairs)						
Metric	**N**	**Mean difference**	**SD**	**Upper LOA**	**Lower LOA**	**Outliers N (%)**
Heart rate (bpm)	109	0.03	1.24	2.46	− 2.40	8 (7.3)
PR interval (ms)	61	7.39	16.88	40.47	− 25.69	4 (6.6)
QRS duration (ms)	109	− 6.04	15.28	23.91	− 35.99	3 (2.8)
QT interval (ms)	109	− 5.32	25.27	44.21	− 54.85	6 (5.5)
QTc interval (ms)	109	− 6.41	30.57	53.50	− 66.33	2 (1.8)
P amplitude (mV)	61	0.01	0.04	0.08	− 0.07	4 (6.6)
QRS amplitude (mV)	109	0.04	0.07	0.18	− 0.09	6 (5.5)
T amplitude (mV)	109	0.00	0.05	0.10	− 0.09	6 (5.5)

Negative values indicated higher measurements from the 6-lead ECG. LOA was calculated using the mean difference and SD. The upper LOA was defined as the mean difference plus 1.96*SD, and the lower LOA was defined as the mean difference minus 1.96*SD. Cases without identifiable P waves were excluded from PR interval and P amplitude analyses.

*ECG* electrocardiogram, *SD* standard deviation, *LOA* limits of agreement, *bpm* beats per minute, *ms* milliseconds, *mV* millivolts

In line with the Bland–Altman analysis, the distribution of the parameter absolute differences in each category was comparable between the supine and sitting positions (e.g., PR interval absolute difference < 20 ms: supine, 71.4%; sitting, 70.5%) (Table 4).

**Table 4:** Absolute differences between the standard 12-lead and handheld 6-lead ECGs under different acquisition conditions

Supine position				
**Metric**	**N**	** < 5**	**5 ≤ < 10**	** ≥ 10**
Heart rate (bpm)	107	107 (100.0%)	0 (0.0%)	0 (0.0%)
		** < 20**	**20 ≤ < 40**	** ≥ 40**
PR interval (ms)	63	45 (71.4%)	16 (25.4%)	2 (3.2%)
QRS duration (ms)	107	81 (75.7%)	26 (24.3%)	0 (0.0%)
QT interval (ms)	107	61 (57.0%)	35 (32.7%)	11 (10.3%)
QTc interval (ms)	107	57 (53.3%)	33 (30.8%)	17 (15.9%)
		** < 0.05**	**0.05 ≤ < 0.1**	** ≥ 0.1**
P amplitude (mV)	63	45 (71.4%)	14 (22.2%)	4 (6.4%)
QRS amplitude (mV)	107	55 (51.4%)	28 (26.2%)	24 (22.4%)
T amplitude (mV)	107	79 (73.8%)	26 (24.3%)	2 (1.9%)
**Sitting position**				
**Metric**	**N**	** < 5**	**5 ≤ < 10**	** ≥ 10**
Heart rate (bpm)	109	107 (98.2%)	2 (1.8%)	0 (0.0%)
		** < 20**	**20 ≤ < 40**	** ≥ 40**
PR interval (ms)	61	43 (70.5%)	17 (27.9%)	1 (1.6%)
QRS duration (ms)	109	80 (73.4%)	29 (26.6%)	0 (0.0%)
QT interval (ms)	109	67 (61.5%)	30 (27.5%)	12 (11.0%)
QTc interval (ms)	109	60 (55.1%)	33 (30.3%)	16 (14.7%)
		** < 0.05**	**0.05 ≤ < 0.1**	** ≥ 0.1**
P amplitude (mV)	61	50 (82.0%)	10 (16.4%)	1 (1.6%)
QRS amplitude (mV)	109	56 (51.4%)	32 (29.4%)	21 (19.3%)
T amplitude (mV)	109	74 (67.9%)	29 (26.6%)	6 (5.5%)
**Asynchronous pairs**				
**Metric**	**N**	** < 5**	**5 ≤ < 10**	** ≥ 10**
Heart rate (bpm)	96	44 (45.8%)	26 (27.1%)	26 (27.1%)
		** < 20**	**20 ≤ < 40**	** ≥ 40**
PR interval (ms)	54	29 (53.7%)	18 (33.3%)	7 (13.0%)
QRS duration (ms)	96	66 (68.8%)	23 (24.0%)	7 (7.3%)
QT interval (ms)	96	46 (47.9%)	31 (32.3%)	19 (19.8%)
QTc interval (ms)	96	33 (34.4%)	28 (29.2%)	35 (36.5%)
		** < 0.05**	**0.05 ≤ < 0.1**	** ≥ 0.1**
P amplitude (mV)	54	37 (68.5%)	10 (18.5%)	7 (13.0%)
QRS amplitude (mV)	96	22 (22.9%)	24 (25.0%)	50 (52.1%)
T amplitude (mV)	96	54 (56.3%)	22 (22.9%)	20 (20.8%)

For asynchronous analysis, non-simultaneous ECGs were matched post hoc by pairing a supine 12-lead ECG with a sitting 6-lead ECG from the same patient. The comparisons were restricted to pairs with consistent rhythm classifications for both devices. Rhythms that may be affected by premature complexes (APC and VPC) were grouped into the sinus rhythm categories. Cases without identifiable P waves were excluded from PR interval and P amplitude analyses

*ECG* electrocardiogram, *bpm* beats per minute, *ms* milliseconds, *mV* millivolts

### Exploratory analyses under different conditions

In the AF-specific numerical agreement analysis, no ECGs were included in the PR interval or P-wave amplitude assessment, because AF is characterized by the absence of P waves. At both acquisition positions, the distribution of absolute differences for each parameter was consistent with the main analysis. However, smaller differences in the QT/QTc intervals were observed in both the supine and sitting positions (Supplementary Table S2).

For asynchronous analysis, 105 ECG pairs were initially matched. After excluding two ungradable 12-lead ECGs, 103 pairs remained for the diagnostic accuracy evaluation (Fig. 3). Overall accuracy was 97.1% (95% CI, 91.7–100.0), which was lower than in the synchronized analysis, despite classifying premature complexes as sinus rhythm. Ninety-six pairs were included in the numerical agreement assessment. Heart rate was higher (12-lead mean, 74.06 bpm; 6-lead, 78.45 bpm), and QT interval (12-lead mean, 421.35 ms; 6-lead, 413.49 ms) was shorter than 12-lead ECG in the 6-lead ECG. However, QTc intervals remained longer in the 6-lead ECG (12-lead, 462.05 ms; 6-lead, 467.14 ms) (Supplementary Table S1). Across all parameters, greater differences were observed between the 12-lead and 6-lead ECGs than in the synchronized analysis (Table 4).

## Discussion

In this study, we demonstrated that the handheld 6-lead ECG provides measurements comparable to those of the standard 12-lead ECG across different acquisition conditions. The diagnostic accuracy exceeded 99% in both the supine and sitting positions. The misclassification of AFL as AF may be due to the absence of precordial leads in the 6-lead ECG, as AFL can be more clearly identified in leads such as V1 or, in some cases, the inferior leads [15]. Bland–Altman analyses showed small mean differences in numerical ECG parameters, generally within 5–15 ms for PR, QRS, and QT/QTc intervals. The mean difference in QTc was slightly higher in the supine position (5 ms) but remained within the range of measurement variability [16]. The limits of agreement were relatively wide, indicating potential variability at the individual level. However, these findings are comparable to those reported in previous studies of handheld 6-lead ECG devices. For example, Metcalfe et al. reported QTc LOA ranging from − 49.97 to 42.63 ms, and Zaballos et al. reported LOA ranging from − 55.12 to 60.92 ms. In comparison, the LOA observed in our study were − 63.19 to 40.16 ms in the supine position and − 66.33 to 53.50 ms in the sitting position [5, 17]. Other parameters’ differences are also consistent with previous findings, suggesting that they are clinically negligible [2, 6–8, 11, 16, 18, 19]. Collectively, these findings indicate that a 6-lead ECG can serve as a reliable alternative to a 12-lead ECG for rhythm classification and signal-based assessments, in settings where a standard 12-lead ECG is unavailable or impractical.

A key strength of this study was the ECGs were obtained in the same body position for each paired comparison. Although postural changes produced the expected physiological effects (e.g. increased heart rate in the sitting position), the relative differences between the 12-lead and 6-lead ECGs remained consistent across postures (e.g. PR interval mean difference of approximately 10 ms in both supine and sitting positions). This indicates that postural influences affect both devices similarly and, therefore, do not bias the assessment of equivalence between the two systems.

Another strength of our study was that it included patients with underlying arrhythmias. Such populations inherently present higher variability in ECG morphology and rhythm, posing greater challenges for measurement reproducibility and device performance [20]. Demonstrating high concordance between the handheld 6-lead and standard 12-lead ECGs under these conditions therefore provides stronger evidence of robustness and clinical applicability. Importantly, in the AF subgroup, the 6-lead ECG showed consistent agreement despite high beat-to-beat variability. QT and QTc interval differences were slightly smaller in AF cases than in all cases, underscoring the stability of the measurements, even in irregular rhythms.

Our findings also extend the findings of previous studies by addressing the limitations of asynchronous acquisition. Previous validation studies often relied on recordings obtained at different times or positions, which can introduce confounding bias [2, 5–7, 9, 11, 17]. While a limited number of studies have employed simultaneous recordings, these have primarily focused on specific applications or remain preliminary [3, 10]. In our analysis, asynchronous comparisons resulted in larger discrepancies in QT interval measurements, with a higher proportion of cases exceeding a 20 ms difference compared to both supine and sitting synchronous recordings. Although asynchronous comparisons also showed a reversal in the direction of mean differences, this is likely attributable to postural effects, as the QT interval is influenced by heart rate and tends to shorten in the sitting position [21–23]. By using synchronized acquisitions, our study eliminated such temporal and positional bias, thereby providing a clearer assessment of intrinsic differences between the two devices.

This study has several significant clinical implications. HATIV's high diagnostic accuracy and strong numerical agreement with 12-lead ECG, coupled with its portability and ease of use, are valuable tools for expanding cardiac monitoring beyond traditional clinical settings. It can enhance timely access to ECG assessments in community, remote, and hospital-at-home environments, where conventional 12-lead ECGs are often impractical or unavailable [24, 25]. Specifically, its proven efficacy in AF detection and QT interval monitoring highlights its potential for detecting symptomatic paroxysmal arrhythmias. This may provide patients and clinicians an objective basis for additional follow-up visits. However, this study was conducted in a controlled clinical environment, which may differ from real-world ambulatory use. Therefore, further studies are needed to evaluate device performance in self-administered or community-based settings.

Despite its overall favorable performance, the 6-lead ECG inherently has several limitations and requires further research. Unlike 12-lead ECGs, which provide a comprehensive spatial assessment including precordial leads (V1–V6), 6-lead devices primarily capture frontal plane activity. Consequently, they are not designed to detect conditions that rely on precordial lead information, such as acute myocardial infarction, VPC origin localization, Brugada patterns, or left ventricular hypertrophy, and may have limited ability to localize ischemic changes. This structural limitation may also affect QTc prolongation assessment, as the longest QT interval is often observed in precordial leads such as V2 or V3. In addition, small differences in QTc measurements observed in this study are likely within the range of measurement variability, but may still have clinical implications in certain contexts. These differences may also be influenced by relatively increased noise in handheld 6-lead ECG recordings compared to standard 12-lead systems. Therefore, comparisons with other portable ECG modalities, such as single-lead or smartwatch-based devices, represent an important area for future investigation.

### Limitations

Despite these promising results, it is crucial to acknowledge the limitations of this study. First, as a single-center observational study, the generalizability of our findings may be limited, necessitating larger multi-center validation studies. Second, because all recordings were performed in a hospital setting under trained supervision, the quality of the mobile device recordings was maintained consistently. In ambulatory practice, however, recording quality may vary, potentially affecting signal equivalence. Third, in the supine position, an additional electrode was used to simulate the left leg contact for the handheld 6-lead ECG. This setup does not reflect typical real-world usage of handheld devices. Fourth, the difference in sampling rates between the HATIV (250 Hz) and 12-lead ECG (500 Hz) could theoretically affect the detection of high-frequency components, although our results suggest that this did not significantly affect the measured parameters within clinically relevant ranges. Finally, this study evaluated a single commercially available 6-lead ECG device (HATIV), and performance may differ across devices from other manufacturers.

## Conclusions

In conclusion, the handheld 6-lead ECG demonstrated excellent agreement with the standard 12-lead ECG in terms of diagnostic accuracy and numerical ECG parameters under synchronized conditions. Its reliable performance in arrhythmic cohorts further supports its clinical utility. However, asynchronous acquisition was associated with larger discrepancies, emphasizing the importance of synchronized recordings in validation studies and the need for caution when interpreting measurements obtained under different acquisition settings. Future multi-center studies with larger and more diverse populations are warranted to confirm these findings and explore the broader applications of handheld 6-lead ECGs in clinical practice.

## Supplementary Information

Supplementary Material 1.

## Data Availability

The data on which this article is based are available in the article itself and in the supplementary material. Due to intellectual property constraints, sharing the code is restricted. However, restricted access to statistical analysis for verified researchers may be provided upon request for research purposes.

## Abbreviations

AF: Atrial fibrillation
AFL: Atrial flutter
APC: Atrial premature complex
bpm: Beats per minute
BSA: Body surface area
CI: Confidence interval
ECG: Electrocardiogram
LOA: Limits of agreement
ms: Milliseconds
mV: Millivolts
NPV: Negative predictive value
PPV: Positive predictive value
SD: Standard deviation
VPC: Ventricular premature complex
