# Supplementary material for: Position-wise comparison of handheld 6-lead ECG versus the standard 12-lead ECG in patients with arrhythmia: comparative study
Source: BMC Cardiovasc Disord. 2026 Jun 8;26:689. doi: 10.1186/s12872-026-05971-x (PMC13463521; doi:10.1186/s12872-026-05971-x)
Supplement: Supplementary file 1 [file 12872_2026_5971_MOESM1_ESM.docx]

# Supplementary File

## Supplementary Figure S1. Asynchronous pairing of 12-lead and 6-lead ECGs.

For exploratory analysis, 12-lead ECGs acquired in the supine position were matched post hoc with 6-lead ECGs acquired in the sitting position to evaluate postural effects.

ECG, electrocardiogram

## Supplementary Table S1. Descriptive statistics of numeric parameters from simultaneously acquired ECG pairs in different acquisition positions.

|  | **Supine**  **(107 pairs)** | | **Sitting**  **(109 pairs)** | | **Asynchronous**  **(96 pairs)** | |
| --- | --- | --- | --- | --- | --- | --- |
| **Metric** | **12-lead  (Mean ± SD)** | **6-lead  (Mean ± SD)** | **12-lead  (Mean ± SD)** | **6-lead  (Mean ± SD)** | **12-lead  (Mean ± SD)** | **6-lead  (Mean ± SD)** |
| **Heart rate (bpm)** | 74.57 ± 17.71 | 74.54 ± 17.70 | 78.43 ± 19.44 | 78.40 ± 19.41 | 74.06 ± 17.15 | 78.45 ± 18.18 |
| **PR interval (ms)** | 191.12 ± 34.94 | 179.06 ± 39.33 | 189.41 ± 33.15 | 182.02 ± 34.35 | 191.61 ± 35.55 | 177.91 ± 32.10 |
| **QRS duration (ms)** | 103.12 ± 25.09 | 109.5 ± 24.60 | 101.01 ± 24.17 | 107.05 ± 24.43 | 103.65 ± 26.66 | 108.24 ± 24.02 |
| **QT interval (ms)** | 418.37 ± 55.18 | 428.71 ± 58.33 | 408.77 ± 54.10 | 414.1 ± 54.54 | 421.35 ± 54.91 | 413.49 ± 53.18 |
| **QTc interval (ms)** | 460.09 ± 50.10 | 471.61 ± 54.75 | 460.23 ± 48.79 | 466.64 ± 54.69 | 462.05 ± 50.52 | 467.14 ± 54.22 |
| **P amplitude (mV)** | 0.17 ± 0.06 | 0.18 ± 0.06 | 0.19 ± 0.05 | 0.18 ± 0.05 | 0.16 ± 0.05 | 0.18 ± 0.05 |
| **QRS amplitude (mV)** | 0.99 ± 0.38 | 0.95 ± 0.38 | 1.00 ± 0.36 | 0.96 ± 0.35 | 1.00 ± 0.36 | 0.97 ± 0.34 |
| **T amplitude (mV)** | 0.23 ± 0.10 | 0.24 ± 0.09 | 0.25 ± 0.11 | 0.25 ± 0.11 | 0.23 ± 0.11 | 0.25 ± 0.11 |

When a P wave was absent in one or more beats, corresponding ECG was excluded from the PR interval and P amplitude comparisons. In the supine position, 63 out of the 107 pairs were used from PR interval and P amplitude comparison. In the sitting position, 61 out of the 109 pairs were used.

ECG, electrocardiogram; SD, standard deviation; bpm, beats per minute; ms, milliseconds; mV, millivolts

## Supplementary Table S2. Absolute differences between the standard 12-lead and handheld 6-lead atrial fibrillation ECGs in different acquisition positions.

| **Supine position** | | | | |
| --- | --- | --- | --- | --- |
| **Metric** | **N** | **<5** | **5≤ <10** | **≥10** |
| **Heart rate (bpm)** | 33 | 33 (100.0%) | 0 (0.0%) | 0 (0.0%) |
|  |  | **<20** | **20≤ <40** | **≥40** |
| **PR interval (ms)** | 0 | - | - | - |
| **QRS duration (ms)** | 33 | 26 (78.8%) | 7 (21.2%) | 0 (0.0%) |
| **QT interval (ms)** | 33 | 22 (66.7%) | 7 (21.2%) | 4 (12.1%) |
| **QTc interval (ms)** | 33 | 20 (60.6%) | 8 (24.2%) | 5 (15.2%) |
|  |  | **<0.05** | **0.05 ≤ <0.1** | **≥0.1** |
| **P amplitude (mV)** | 0 | - | - | - |
| **QRS amplitude (mV)** | 33 | 17 (51.5%) | 9 (27.3%) | 7 (21.2%) |
| **T amplitude (mV)** | 33 | 25 (75.8%) | 7 (21.2%) | 1 (3.0%) |
| **Sitting position** | | | | |
| **Metric** | **N** | **<5** | **5≤ <10** | **≥10** |
| **Heart rate (bpm)** | 37 | 36 (97.3%) | 1 (2.7%) | 0 (0.0%) |
|  |  | **<20** | **20≤ <40** | **≥40** |
| **PR interval (ms)** | 0 | - | - | - |
| **QRS duration (ms)** | 37 | 30 (81.1%) | 7 (18.9%) | 0 (0.0%) |
| **QT interval (ms)** | 37 | 27 (73.0%) | 7 (18.9%) | 3 (8.1%) |
| **QTc interval (ms)** | 37 | 23 (62.2%) | 11 (29.7%) | 3 (8.1%) |
|  |  | **<0.05** | **0.05≤ <0.1** | **≥0.1** |
| **P amplitude (mV)** | 0 | - | - | - |
| **QRS amplitude (mV)** | 37 | 18 (48.7%) | 13 (35.1%) | 6 (16.2%) |
| **T amplitude (mV)** | 37 | 26 (70.3%) | 11 (29.7%) | 0 (0.0%) |

Only ECG pairs classified as atrial fibrillation in both the 12-lead and 6-lead recordings were included in the analysis.

ECG, electrocardiogram; bpm, beats per minute; ms, milliseconds; mV, millivolts
